# Clothing for the British Climate

**Published:** 1911-01-07

**Authors:** 


### Clothing for the British Climate.

The correct weight of clothing is regarded by Mr. Cantlie as a most important factor in the maintenance of health, and in the *Journal of Tropical Medicine and Hygiene* he lays down certain rules which he claims to have established by observation. For these islands the normal weight of clothing is one-fourteenth of the body weight—that is, one pound for every stone of the wearer. This standard is commonly adhered to for girl children, but boys, even at tender ages, often wear much less than is required by the rule. Mr. Cantlie goes on to state that the girls of the present day are thriving and growing well, whereas their brothers are too often delicate and weedy in comparison. The cause, he believes, of the alleged male inferiority is insufficient clothing; but if the author were to reduce this series of propositions to syllogisms he would very soon find himself confronted by several obvious fallacies. As he justly says, personal experience is worth a pound of theory, and his own impressions are interesting as far as they go. But whether the conclusions reached are right or wrong—and it is at the least uncertain that they are right—the premisses on which they are founded and the reasoning by which they are reached are so shaky that the profession cannot be expected to assent to them without more definite proofs.

Mr. Cantlie goes on to state that in examining men for appointments in the tropics he finds the weight of clothing worn and the health of the candidates to be in direct proportion. No young man who wears neither under-vest nor drawers winter and summer has ever been found by him to be physically fit. He has for years been looking for an exception to this rule, but has not yet succeeded. Of fifteen young men thus rejected (for reasons of health, of course, not for wearing scanty under-garments) he found that nine had temperatures of 100° F. or more at twelve noon. As he suggests, it is natural to suppose that they wore no underclothes because they felt too hot in them. All these young men denied all knowledge of feverishness, and said that they felt perfectly well. Three of the nine had (persistent) albuminuria. In six cases of underclothed candidates there was cardiac disorder; in two cases a defective aortic valve; and in the others some degree of dilatation-hypertrophy. Several of them had very irritable hearts, the rhythm of which was easily disturbed. The author sums up that a man who has not sense to keep clothes on his back is neither mentally nor physically of much use in this world. Such may be his conviction, but he offers no shadow of proof to support it. Without denying that a certain number of people in these islands are voluntarily underclad, it must be protested that Mr. Cantlie has exaggerated his case to such an extent that he has destroyed his chance of winning a verdict. One extreme is as bad as the other—undue coddling, and reckless exposure to our variable and trying climate. In matters of dress, as in all else, the watchword must be moderation. If men, women, and children, while observing the dictates of hygienic prudence and common-sense in all other directions, wear such clothing as they find most comfortable, sufficiently warm without being burdensome, and best suited for protection from the weather, it may be safely argued that the exact number of pounds which it weighs is of small moment, and that the future of the race may be left to take care of itself.

